# Editorial: Insights in nanobiotechnology 2024/2025: novel developments, current challenges, and future perspectives

**DOI:** 10.3389/fbioe.2026.1795314

**Published:** 2026-02-10

**Authors:** Pilar Rivera Gil, Christian Celia, Gianni Ciofani

**Affiliations:** 1 Integrative Biomaterials and Nanomedicine Lab, Medicine and Life Science Department, Systems Biology and Biomedical Engineering Programme, Universitat Pompeu Fabra, Barcelona, Spain; 2 Department of Pharmacy, University of Chieti-Pescara “Gabriele d’Annunzio”, Chieti, Italy; 3 Smart Bio‐Interfaces Research Unit, Italian Institute of Technology, Pontedera, Italy

**Keywords:** biological clock, nanobiotechnology, organ on chips, photocatalytic systems, smart nanoparticles

Nanobiotechnology is progressing rapidly, and the last few years have brought a noticeable shift in what the community expects as proof. Advances in nanomaterial design, characterization, and experimental models now make it possible to ask more demanding questions: not only whether a nano-enabled intervention works, but why it works, when it fails, and how its performance depends on context. In practice, the field is moving from demonstrations in simplified settings to nanosystems the behaviour of which can be explained mechanistically, reproduced across laboratories, and steered in realistic biological or environmental conditions.

This Research Topic contributes to that broader goal by bringing together forward-looking pieces that highlight both progress and bottlenecks across the nano-bio interface. This edition gathers four articles spanning immunomodulatory nanomaterials (Ceccarelli et al.), photocatalytic nanocomposites for environmental remediation (Ram et al.), computational design frameworks for organ-on-chip systems (Taleban et al.), and quantitative perspectives on biological timing and synchrony (Al-Omari et al.).

Despite Al-Omari et al. is not centred on nanomaterials, the contribution underscores a point that carries directly to nanobiotechnology: time and heterogeneity are not nuisance variables. While the topics range from immune modulation to microphysiology and microbial rhythms, they converge on a common theme: understanding and controlling nano-bio behaviour requires attention to mechanism, microenvironment, and time. In other words, there is no single nano-bio interface. Depending on the question, it might be an immune cell deciding which phenotype to adopt, a microfluidic device setting gradients and forces that shape cell fate, a catalytic surface driving chemical transformation in water, or a population of single cells synchronizing their behaviour over time.

Across these four papers, several challenges emerge that are common to much of nanobiotechnology. Reproducibility and comparability still hinge on variables that are easy to underreport, such as dispersion state in biological media, batch-to-batch variation, and dose definitions that go beyond nominal mass concentration (particle number, surface area, or delivered dose).

Equally, biological relevance is increasingly an engineering constraint. If gradients, mechanics and exposure time are not designed and validated, experimental outcomes can be systematically biased. Translation adds further layers, whether in photocatalysis (energy input, robustness, and by-products under realistic matrices) or in immunomodulation (immune ecosystem effects, safety, and manufacturability). These constraints are not administrative afterthoughts, they are design parameters that can and should be considered early.

Looking forward, three priorities stand out. First, mechanism-first nanobiotechnology: linking physicochemical features to causal biological or chemical pathways, supported by time-resolved, multi-parameter phenotyping and perturbations that discriminate between alternative explanations. Second, predictive design loops: integrating simulation, controlled microenvironments, and data to generate and prioritise hypotheses, and then stress-testing those hypotheses experimentally with explicit uncertainty and sensitivity analyses; modelling is most valuable when it guides which experiments to do next, how to interpret discordant readouts, and how to define the boundary conditions under which an effect is expected to hold. Third, responsible innovation by design: sustainability, life-cycle considerations, and safety-by-design treated alongside performance, together with regulatory-ready documentation of composition, stability, and quality attributes; the aim is not to slow discovery, but to avoid predictable failure modes and accelerate credible translation.

Taken together, these papers reinforce a practical direction for the field: design choices that can be defended mechanistically, experimental models treated as controllable systems, and earlier engagement with translation constraints so that promising concepts are not lost for avoidable reasons. As with any Research Topic, the four articles are not meant to be exhaustive. Instead, they illustrate how the nano-bio interface can be approached as a set of measurable, testable relationships that span materials, models, and time. We hope this Research Topic will stimulate discussion, inspire collaborations, and help sharpen the evidence standards that move nanobiotechnology from demonstration to dependable application.

Prof. Ciofani is a shareholder of Kidaria Bioscience SRL, but declares no competing interests related to the article Research Topic.

